# Relationship between Body Composition and Specific Motor Abilities According to Position in Elite Female Soccer Players

**DOI:** 10.3390/ijerph20021327

**Published:** 2023-01-11

**Authors:** Mima Stanković, Ilma Čaprić, Dušan Đorđević, Stefan Đorđević, Adem Preljević, Admira Koničanin, Džejla Maljanović, Hamza Nailović, Iso Muković, Igor Jelaska, Goran Sporiš

**Affiliations:** 1 Faculty of Sport and Physical Education, University of Niš, 18000 Niš, Serbia; mima.stankovic974@gmail.com (M.S.); stefan-djordjevic1@hotmail.com (S.Đ.); 2 Faculty of Sport and Physical Education, State University of Novi Pazar, 36300 Novi Pazar, Serbia; capricilma.np@gmail.com (I.Č.); apreljevic@np.ac.rs (A.P.); akonicanin@np.ac.rs (A.K.); dzejla0000@gmail.com (D.M.); hamza.nailovic00@gmail.com (H.N.); isomukovic2019@gmail.com (I.M.); 3 Faculty of Kinesiology, University of Split, 21000 Split, Croatia; jelaska@kifst.hr; 4 Faculty of Kinesiology, University of Zagreb, 10110 Zagreb, Croatia; goran.sporis@kif.unizg.hr

**Keywords:** football, agility, endurance, strength, fats, muscle mass, body fluids

## Abstract

Contemporary top-division soccer is characterized by high-intensity activity throughout the entire match, which also requires high levels of a wide range of the players’ functional and motor abilities. Furthermore, motor and functional requirements vary in relation to the players’ position on the pitch. In view of the above, the objective of this study was to determine any differences in body composition and specific motor abilities in relation to position. Twenty elite female soccer players (age: 20.90 ± 3.70 years; height: 166.95 ± 5.83 cm; weight: 58.97 ± 7.50 kg; training experience: 9.50 ± 4.11 years) were recruited for the purpose of this study. Based on their position within the team, the players were divided into three groups: defenders (N-7), midfielders (N-6), and forwards (N-7). The instruments used included the InBody770 (for body composition assessment), Optojump and Polar for the assessment of specific motor abilities. The results obtained indicate a strong link between the parameters body composition and specific motor abilities; however, the level of significance varies, as do the variables concerning specific motor abilities and body composition in relation to the players’ position on the pitch. In accordance with these results, coaches and others working in the soccer industry should be apprised of the necessity of a tailored approach when it comes to planning the development of specific motor abilities, as well of with the importance of balanced body composition as prerequisites for achieving top results.

## 1. Introduction

In the broad domain of physical education and sport, soccer is simultaneously a physical and cognitive activity that has proven itself irresistibly popular with people all over the world [1]. Contemporary top-level soccer is marked by high-intensity activity throughout the entire match, also requiring a high level of a wider array of players’ functional and motor abilities. Without exception, all motor and functional abilities are important to success in soccer; however, the dominant abilities are speed, explosive strength, endurance, agility, and coordination [2].

Given the importance of motor abilities, not only for the quality of the game but also for the individual, many studies have tried to determine the factors that affect the quality of motor abilities, and the extent thereof, whereas success in soccer was to a considerable extent dependent on various anthropological dimensions [3,4]. Furthermore, there were diverse opinions about the body composition and its effect on situational-motor abilities, where Ismaili et al. [5] have presented greatest adverse, while Stanković et al. [6] have showed the result maintained. Likewise, they should not disregard the fact that bone mass comprises 18–20% of the total, muscles comprise 50–53%, while fatty tissue mass is 8–10% in soccer players [7].

Based on the structure of competitive activity, as well as based on its overall character, it is possible to define a hypothetical model of anthropological characteristics for soccer players playing in different positions [8]. Based on this hypothetical model pertaining to players’ various positions, it is possible to devise a classification into two groups of players, based on the player formation most frequently applied in contemporary soccer; namely, the 4-4-2 formation, as well as based on shared anthropological characteristics, the structure and character of activities performed during a match. Thus, the first group comprises players with a somewhat lesser engagement in the game (the goalkeeper, two center defenders, and two forwards), while the second group includes players with more extensive game engagement (two wing backs and four midfielders). Based on the model of base motor domain structure, a hypothetical model of base motor abilities for specific groups of player positions has been defined, including a selection of base motor tests for their assessment [7].

The hypothetical model of base and specific motor abilities for the group with a relatively lesser engagement in the game (goalkeeper, two center defenders, and two forwards) yields players with exceptional explosive and repetitive strength, speed, agility, and specific explosive strength. The hypothetical model of base and specific motor abilities for the group with the more extensive game engagement (two wingbacks, and four midfielders) presents a group of exceptional endurance, coordination, flexibility, and a highly developed specific speed, specific precision and specific coordination [9].

There are differences between soccer players in accordance with the position they play in the team. These differences correspond to the different tasks they perform during the game, and, accordingly, the training program ought to account for tasks specific to each position within the team [10]. Based on morphological characteristics measurements, Tomić et al. [7] have reported significant differences between the five sub-samples of positions within the game, for the multiple parameters measured (body height, body mass, leg length, foot length, pelvic width, knee diameter, ankle diameter, mean chest circumference, lower leg circumference, and back skinfold). In addition, the same study has reported no difference for couple of parameters measured (thigh circumference, upper arm skinfolds, suprailiac skinfold and thigh skinfold).

Unlike men’s soccer, women’s soccer has yet to reach its male counterpart’s level of popularity and professionalism. Moreover, although the data on the increase in the number of registered female players indicates both a significant growth and popularization of the sport, the relevant research and studies into women’s soccer remain scarce [11,12]. Hence, we have aimed to identify the relationship between body composition and specific motor abilities, according to the player’s position. The significance of this study lies in the fact that the results obtained herein will enable coaches to identify the player characteristics that can facilitate their decision process when selecting for a specific team position. In addition, we have hypothesized that the study results will show strong correlation regarding body composition and motor abilities.

## 2. Materials and Methods

### 2.1. Participant Sample

Twenty elite female soccer players (age: 20.90 ± 3.70 years; height: 166.95 ± 5.83 cm; weight: 58.97 ± 7.50 kg; training experience: 9.50 ± 4.11 years) were recruited for the purpose of this study. The data were collected during the preparatory period of 2020. The team that participated in the research plays in the elite ranking league in Serbia and was playing in the playoffs at the time of measurement. The inclusion criteria for participant recruitment were as follows: at least 5 years of training experience, active training history (more than 7 h per week) in the last 12 months, and did not have any existing injury or medical condition that would compromise the participation. Furthermore, based on the players’ position in the team, the included participants were divided into the following groups: defenders (N-7), midfielders (N-6) and forwards (N-7).

The players were voluntarily participating in the testing, although written consent was also obtained. Furthermore, this study was approved by the Faculty of Sport and Physical Education, University of Niš (code: 04-784-2) and followed the ethical standards of The Declaration of Helsinki for the study of humans.

### 2.2. Measuring Instruments (Testing Procedure)

As part of the testing, the players were asked to complete a battery of tests aimed at the assessment of specific motor abilities, as well as tests for determining body composition. Height was measured using the Martin anthropometer, whose validity has been presented elsewhere (Lacy and Williams, 2018) [13]. The authors gave the participants instructions on how to prepare for this measurement one day before testing. Participants did not consume food or beverages before the test in the morning.

Using the InBody instrument for determining body composition, the following variables were obtained: BH—body height, BW—body weight, BMI—body mass index, BFkg—body fat, BF%—body fat percentage, FMI—fat mass index, NFBMkg—non-fatty body mass, PNTM—percentage of non-fatty body mass, MMkg—muscle mass, and PMM—muscle mass percentage. The InBody 770 (InBody Co., Seoul, Republic of Korea) was used for the InBody test, whereas instrument validity has been presented elsewhere [14]. Additionally, there has been research where this instrument was applied, and where the study participants were also senior-group female soccer players [15,16]. The formula (fat mass/height^2^) was used to calculate the Fat Mass Index (FMI). This formula was also used in a previously reported study (Kelly, Wilson, and Heymsfield, 2009) [17].

The battery of tests for the assessment of specific motor abilities consisted of the following test groups: speed assessment tests: the 0 to 20 m run test (s20), used in the study of motor abilities of senior female athletes in various sports [18]. Agility tests: “505“ (a505), the 9-6-3-6-9 test, with a 180° turn (a96369), used in papers researching seniors [19]. Zig-zag running without the ball (CIK-CAK), and zig-zag running with the ball (CIK-CAKLop), applied in the study of the elite female soccer players of the Montenegrin national team [20]; the 4 × 5 m running test without the ball (a45), the 4 × 5 m running test with the ball (a45Lop), as used in studies of junior soccer players [21]. The yo–yo test of speed endurance (YY) was measured using the Polar (Team pro polar, professorintie 5, Kempele, Finland), whose validity was determined in studies examining sprint in elite female soccer players [22,23]. Next was the agility *t*-test (*t*-test) [24]. Leg explosive strength was tested by means of the following tests: squat jump (SJ), countermovement jump (CMJ), countermovement jump with arm swing (CMJa).

Time was measured using photocell devices (Witty, Microgate, Bolzano, Italy) with a measurement precision of 1/100, as well as cones to mark the distances and balls in those exercises which required their use. In the following tests: the squat jump (SJ), countermovement jump (CMJ), and maximum countermovement jump (CMJa), the Optojump was used (Optojump, Microgate, Bolzano, Italy). The above-mentioned tests have also been used in the numerous studies (Lockie, Moreno, Lazar, Orjalo, Giuliano, et al., 2018; Sjökvist, Laurent, Richardson, Curtner-Smith, Holmberg, et al., 2013; Lockie, Moreno, Lazar, Orjalo, Giuliano, Risso, 2018) [25,26,27].

### 2.3. Statistical Analysis

The results obtained in the study were analyzed using the statistical software SPSS, version 20. For all data obtained during the measurements the basic central and distribution parameters were calculated (Arithmetic Mean, minimum and maximum value and standard deviation).

In order to determine whether there is a link between the parameters of body composition and those of the motor abilities of the senior female soccer players, correlation analysis was used (the Pearson correlation coefficient).

## 3. Results

On the total participants’ sample, the distribution normality shows that only two parameters of body composition do not have normal distribution (NFBMkg *p* = 0.028; MMkg *p* = 0.045), in regard to all nine analyzed parameters, Table 1. Likewise, only one specific motor ability parameter (a96369 *p* = 0.000) shows the same, in regard to all 12 analyzed parameters, Table 2.

Based on the presented values in Table 3, a statistically significant positive correlation between the parameters may be singled out: CMJ and PNTM (r = 0.49; *p* = 0.03); CMJ and PMM (r = 0.48; *p* = 0.03); SJ and PNTM (r = 0.56; *p* = 0.01); SJ and PMM (r = 0.64; *p* = 0.00); a45lop and BFkg (r = 0.46; *p* = 0.04) and a45lop with FMI (r = 0.49; *p* = 0.03). Moreover, a statistically significant negative correlation between the parameters was obtained: SJ and BF% (r = −0.53; *p* = 0.02); s505 and PMM (r = −0.47; *p* = 0.04) and YY with FMI (r = −0.50; *p* = 0.02). Obtained results in this way indicate that a statistically significant positive correlation present between the parameters of the explosive strength and non-fat and muscle mass. It was also spotted between one variable for the estimation of the frontal agility and the parameters of the fat body mass and FMI. A statistically significant negative correlation among one variable of the explosive strength and the percentage of body fat mass and one variable for the estimation of the frontal agility and percentage of muscle mass as well as the endurance and fat mass index.

Based on the values obtained in the Table 4, a statistically significant positive correlation among parameters may be noticed: CMJ and PNTM (r = 0.83; *p* = 0.02); CMJ and PMM (r = 0.84; *p* = 0.02). The results obtained in this way show that a statistically significant positive correlation is present at forwards only between one parameter for the estimation of the explosive strength of lower extremities and percentages of fat and muscular body mass.

Based on the values obtained in the Table 5, a statistically significant positive correlation between the parameters may be singled out: a96369 and BF% (r = 0.88; *p* = 0.02); a96369 and FMI (r = 0.87; *p* = 0.02); a45 and BF% (r = 0.91; *p* = 0.01) and a45 with FMI (r = 0.83; *p* = 0.04). Moreover, a statistically significant negative correlation between the following parameters was obtained: CMJa and NFBMkg (r = 0.95; *p* = 0.00); CMJa and MMkg (r = −0.97; *p* = 0.00); s505 and PMM (r = −0.84; *p* = 0.03); a96369 and PNTM (r = −0.88; *p* = 0.02); a96369 and PMM (r = −0.88; *p* = 0.02); a45 and PNTM (r = −0.91; *p* = 0.01); a45 lop and MMkg (r = −0.81; *p* = 0.5) and a45 with PMM (r = −0.93; *p* = 0.01). Obtained results in this way indicate that a statistically significant positive correlation in midfielders is present between the fat percentage and fat mass index with two parameters for the frontal agility evaluation. Negative and statistically significant correlation was spotted between one parameter for the evaluation of the explosive strength of lower extremities and the values of non-fat and muscle mass in kilograms. In addition, a statistically significant negative correlation was noticed between four parameters for the estimation of the agility and the percentage of the non-fat and muscle body mass.

Based on the presented values in Table 6, a statistically significant positive correlation between the parameters may be singled out: CMJ and PNTM (r = 0.85; *p* = 0.02); CMJ and PMM (r = 0.81; *p* = 0.03); CMJa and BH (r = 0.80; *p* = 0.03); CMJa and NFBMkg (r = 0.80; *p* = 0.03); CMJa and MMkg (r = 0.79; *p* = 0.04); SJ and PMM (r = 0.76; *p* = 0.05); s505 and BW (r = 0.78; *p* = 0.04); s505 and BMI (r = 0.84; *p* = 0.02); s505 and BFkg (r = 0.85; *p* = 0.02); s505 and BF% (r = 0.87; *p* = 0.01); s505 and FMI (r = 0.85; *p* = 0.02); *t*-test and FMI (r = 0.93; *p* = 0.01); CIKCAK and FMI (r = 0.91; *p* = 0.01); CIKCAKLop and FMI (r = 0.84; *p* = 0.04); a45lop and BMI (r = 0.87; *p* = 0.01); a45lop and BFkg (r = 0.87; *p* = 0.01); a45lop and BF% (r = 0.92; *p* = 0.00); a45lop and FMI (r = 0.92; *p* = 0.00) and YY with PMM (r = 0.81; *p* = 0.03). Moreover, a statistically significant negative correlation between the parameters was obtained: s505 and PMM (r = −0.77; *p* = 0.04); a96369 and PMM (r = −73; *p* = 0.04); a45lop and PMM (r = −0.85; *p* = 0.02); YY and BW (r = −89; *p* = 0.01); YY and BMI (r = −0.90; *p* = 0.01); YY and BFkg (r = −0.92; *p* = 0.00); YY and BF% (r = −0.91; *p* = 0.01); YY and FMI (r = −0.89; *p* = 0.01) and YY with MMkg (r = −0.77; *p* = 0.04). Obtained results in this way indicate that a statistically significant positive correlation at defenders is present between the parameters of the explosive strength and non-fat and mass body. Moreover, it was spotted between sixth variables for the estimation of the frontal agility and the parameters of the fat body mass and BMI and fat mass index, as well as between the parameters of endurance and percentage of the body mass. A statistically significant negative correlation among three variables of the frontal agility and the percentage of the muscle mass was noted, as well as the endurance and fat body mass, fat mass index.

## 4. Discussion

The objective of the paper was to establish the differences in body composition and specific motor abilities, dependent on the position within the team. The obtained results indicate a strong correlation between the parameters body composition and specific motor abilities. However, the level of significance varies, as do the variables concerning specific motor abilities and body composition in relation to the players’ position on the pitch.

Correlation results for female forwards participating in the study indicate a statistically significant correlation in CMJ with PNTM and PMM, corroborating the findings of similar earlier studies [28]. However, another study [29] did not find correlation between specific parameters. Results thus obtained indicate that there is correlation between explosive leg power, one of the most characteristic motor abilities for this position, and the percentage of fat-free body mass and percentage of muscle mass. In that regard, players in this position are characterized by high-repetition, high-intensity, short-duration activities, such as short sprints, where explosive power is paramount [30]. Moreover, forwards tend to have higher muscle mass percentages compared to players in other positions [10].

Correlation analysis yielded the correlation in CMJa with PNTM and MMkg in midfielders. Likewise, high negative correlation was found between the s505 and PMM, along with a96369 and BF%, a96369 and PNTM, and a96369 and PMM. In regard to our given results for the midfielders, some previous studies are in accordance with ours [29,31]. What is more, correlation between the motor abilities and body composition was also found in a45 and BF%, a45 and PNTM, as well as with a45 and PMM. The final set of parameters for which negative correlation was found were a45Lop and MM. The data collected indicate that, as with forwards, players with a higher ratio of fat-free body mass achieve better results in tests assessing explosive power. The characteristic correlation for this group of subjects, the players at the midfield line, is a negative correlation between tests of agility and the percentage of body fat, as well as between fat-free body mass and the percentage of muscle mass. Midfielders are widely known to be characterized by greater agility compared to the other positions on the team [32]. Hence, this study has demonstrated that higher percentages of body fat, fat-free body mass, and muscle mass have an adverse effect on the degree of agility in female midfielders. Based on the results obtained, we are emphasizing that midfielders should not have excessive muscle mass but should also not have a very high body fat percentage.

As far as defenders are concerned, our results indicate statistically significant correlation between parameters CMJ with PNTM and PMM; CMJa with BH, NTM, and MM. Strong correlations were also found between parameters SJ and PMM; between parameters a505 and BW, BMI, BFkg, BF%, and PMM. Based on the findings where significant correlation was found between the parameter explosive power on the one hand, and muscle mass percentage and fat-free body mass percentage on the other, our results are in accordance with the previously published study [28].

In defenders, the results also indicate a correlation between a96369 and PMM, as well as in a45Lop with BMI, BFkg, BF% and PMM. Agility is one of the most important motor abilities, and such results indicate that agility is significantly affected by muscle mass percentage, BMI, and body fat percentage [33].

The final set of parameters for which correlation was found, a negative one, was between parameters YY with BW, BMI, BFkg, BF%, MM, whereas positive was identified between YY and PMM. The final correlations ascertained are related to soccer players’ endurance and body composition relation. The results obtained indicate that body mass, BMI, and body fat percentage have a negative effect on endurance tests, whereas muscle mass percentage has a positive effect on the test of endurance [34]. In addition, our results support the results of study [9], which revealed that elite level midfielders had lower fa mass percentage and higher VO2max than other positional roles. To some extent, this may be due to the unique match demands of each playing position, which are determined by a variety of contextual factors [9]. Consequently, each playing position has a “unique” physiological background [35].

To begin, it should be noted that the sample size was small and the data were limited to a specific group of soccer players, so additional research to confirm the current findings would be beneficial. Female soccer players have characteristics that make it difficult to extrapolate our findings to other sports. This study did not consider variables related to the female athletes’ genotype nor menstrual cycle that could affect the result. Future research should extend these findings to other age groups, competitive levels, and larger samples to see if the results are similar.

## 5. Conclusions

Based on the results obtained in this study, it can be concluded that there is a statistically significant association between all the parameters of body composition and specific motor abilities, as well as between body composition and endurance. In accordance with the results, the significance of this study lies primarily in providing an insight into athletes’ status, and enabling coaches to program and plan the training process with greater precision, in addition to facilitating the selection of the most appropriate player position based on certain body composition characteristics. From the perspective of the player, the potential contribution of this study lies in identifying precisely and efficiently those abilities that may require enhancement in order to achieve top results in soccer.

Finally, the insights this study has yielded have opened avenues for future research, with opportunities for other researchers working in this area to provide answers to some of the questions arising from the results of this study.

## 6. Practical Applications

The current study demonstrated a statistically significant relationship between all body composition parameters and specific motor abilities, as well as between body composition and endurance. Because of the unique characteristics of each player position, this fact can be extremely important, especially given the importance of performance improvement. Given the rapid growth of women’s soccer, this information may be of interest to coaches and sports scientists.

## Figures and Tables

**Table 1:** Body composition parameters’ descriptives.

	Min.	Max.	M ± SD	K–S (Sig.)	S–W (Sig.)
BH	158.00	178.00	166.85 ± 5.90	0.200	0.335
BW	44.00	72.20	59.02 ± 7.88	0.200	0.844
BMI	17.40	25.70	21.23 ± 2.10	0.200	0.981
BFkg	6.80	24.00	14.73 ± 4.42	0.200	0.780
BF%	15.40	34.10	24.37 ± 4.87	0.160	0.434
FMI	2.69	8.00	5.26 ± 1.48	0.480	−0.676
NFBMkg	31.90	51.40	44.10 ± 5.22	0.028	0.129
PNTM	59.51	89.49	75.12 ± 6.75	0.200	0.891
MMkg	20.30	28.40	24.56 ± 2.63	0.045	0.055
PMM	35.95	50.09	41.86 ± 3.20	0.200	0.623

Legend: Min—minimal value; Max—maximal value; M—mean; SD—standard deviation; BH—body height; BW—weight; BMI—body mass index; BFkg—body fat; BF%—body fat percentage; FMI—fat mass index; NFBMkg—non-fatty body mass; PNTM—non-fatty body mass percentage; MMkg—muscle mass; PMM—muscle mass percentage; K–S (Sig.)—Kolmogorov–Smirnov Z test significance; S–W (Sig.)—Shapiro–Wilk test significance.

**Table 2:** Specific motor ability parameters descriptives.

	Min.	Max.	M ± SD	K–S (Sig.)	S–W (Sig.)
CMJ	17.30	28.80	23.51 ± 3.46	0.200	0.426
CMJa	21.60	33.80	28.03 ± 3.30	0.200	0.816
SJ	15.50	28.20	22.30 ± 3.47	0.182	0.725
s20	3.37	3.99	3.66 ± 0.138	0.200	0.592
a505	2.55	3.17	2.81 ± 0.18	0.200	0.264
a96369	1.36	9.91	8.91 ± 1.81	0.000	0.000
*t*-test	10.59	12.70	11.56 ± 0.49	0.200	0.891
CIKCAK	4.94	5.81	5.45 ± 0.23	0.200	0.507
CIKCAKLop	6.33	9.17	7.44 ± 0.68	0.200	0.583
a45	6.12	7.57	6.73 ± 0.33	0.200	0.700
a45Lop	7.66	11.20	8.58 ± 0.87	0.200	0.900
YY	1600.0	3600.0	2774.0 ± 631.77	0.200	0.135

Legend: Min—minimal value; Max—maximal value; M—mean; SD—standard deviation; CMJ—countermovement jump; CMJa—countermovement jump with arms swing; SJ—squat jump; s20—0–20 m run test; a505—agility test “505“; a96369—the 9-6-3-6-9 with a 180° turn; *t*-test—agility *t*-test; CIK-CAK—zig-zag running without the ball; CIK-CAK Lop—zig-zag running with the ball; a45—4 × 5 m running test without the ball; a45Lop—4 × 5 m running test with the ball; YY—Yo-Yo test of speed endurance; K–S (Sig.)—Kolmogorov–Smirnov Z test significance; S–W (Sig.)—Shapiro–Wilk test significance.

**Table 3:** Pearson’s correlation between body composition and specific parameters of motor ability.

		BH	BW	BMI	BFkg	BF%	FMI	NFBMkg	PNTM	MMkg	PMM
CMJ	Pearson Correlation	−0.23	−0.28	−0.13	−0.36	−0.39	−0.32	0.04	0.49	−0.01	0.48
	Sig. (2-tailed)	0.33	0.23	0.59	0.12	0.09	0.17	0.87	0.03	0.97	0.03
CMJa	Pearson Correlation	0.22	0.29	0.30	0.27	0.27	0.29	0.28	−0.18	0.28	−0.14
	Sig. (2-tailed)	0.37	0.23	0.21	0.26	0.26	0.23	0.24	0.46	0.24	0.56
SJ	Pearson Correlation	−0.08	−0.21	−0.13	−0.43	−0.53	−0.43	0.20	0.56	0.21	0.64
	Sig. (2-tailed)	0.73	0.38	0.60	0.06	0.02	0.06	0.41	0.01	0.38	0.00
s20	Pearson Correlation	0.20	0.15	−0.03	0.25	0.33	0.21	−0.05	−0.30	−0.11	−0.44
	Sig. (2-tailed)	0.39	0.52	0.89	0.29	0.15	0.37	0.84	0.20	0.64	0.05
s505	Pearson Correlation	0.04	0.20	0.21	0.34	0.42	0.38	0.02	−0.29	−0.07	−0.47
	Sig. (2-tailed)	0.88	0.39	0.38	0.15	0.06	0.10	0.93	0.22	0.78	0.04
a96369	Pearson Correlation	0.08	0.27	0.31	0.25	0.20	0.25	0.15	−0.19	0.20	−0.17
	Sig. (2-tailed)	0.73	0.27	0.20	0.30	0.41	0.30	0.55	0.43	0.42	0.48
*t*-test	Pearson Correlation	0.30	0.43	0.34	0.43	0.39	0.39	0.35	−0.16	0.26	−0.37
	Sig. (2-tailed)	0.21	0.07	0.15	0.07	0.10	0.10	0.14	0.53	0.28	0.12
CIKCAK	Pearson Correlation	0.17	0.38	0.39	0.35	0.34	0.37	0.26	−0.23	0.25	−0.33
	Sig. (2-tailed)	0.50	0.11	0.10	0.14	0.15	0.12	0.28	0.35	0.30	0.18
CIKCAKLop	Pearson Correlation	−0.14	−0.07	−0.09	0.03	0.07	0.05	−0.22	−0.16	−0.26	−0.21
	Sig. (2-tailed)	0.57	0.76	0.71	0.92	0.76	0.83	0.36	0.51	0.28	0.38
a45	Pearson Correlation	0.11	0.36	0.37	0.39	0.42	0.42	0.20	−0.31	0.17	−0.43
	Sig. (2-tailed)	0.64	0.12	0.11	0.09	0.07	0.07	0.41	0.18	0.48	0.06
a45Lop	Pearson Correlation	0.05	0.36	0.44	0.46	0.44	0.49	0.18	−0.28	0.15	−0.40
	Sig. (2-tailed)	0.84	0.12	0.05	0.04	0.05	0.03	0.45	0.24	0.54	0.08
YY	Pearson Correlation	−0.23	−0.43	−0.38	−0.51	−0.52	−0.50	−0.26	0.31	−0.20	0.49
	Sig. (2-tailed)	0.33	0.06	0.09	0.02	0.02	00.02	0.27	0.19	0.41	0.03

**Table 4:** Pearson’s correlation between body composition and specific parameters of motor ability in soccer players of the forwards.

		BH	BW	BMI	BFkg	BF%	FMI	NFBMkg	PNTM	MMkg	PMM
CMJ	Pearson Correlation	−0.09	−0.38	−0.29	−0.51	−0.65	−0.58	0.16	0.83	0.18	0.84
	Sig. (2-tailed)	0.86	0.40	0.53	0.25	0.11	0.17	0.73	0.02	0.71	0.02
CMJa	Pearson Correlation	0.26	0.18	0.29	0.04	−0.08	0.00	0.51	0.27	0.50	0.29
	Sig. (2-tailed)	0.57	0.71	0.53	0.93	0.86	0.99	0.24	0.55	0.26	0.53
SJ	Pearson Correlation	−0.28	−0.37	−0.07	−0.43	−0.46	−0.39	0.08	0.67	0.12	0.71
	Sig. (2-tailed)	0.54	0.41	0.88	0.34	0.30	0.38	0.87	0.10	0.80	0.08
s20	Pearson Correlation	0.21	0.31	−0.02	0.32	0.32	0.27	−0.11	−0.59	−0.15	−0.64
	Sig. (2-tailed)	0.65	0.49	0.97	0.49	0.48	0.56	0.82	0.16	0.75	0.12
s505	Pearson Correlation	−0.16	−0.26	−0.46	−0.22	−0.15	−0.25	−0.39	−0.02	−0.41	−0.07
	Sig. (2-tailed)	0.73	0.57	0.30	0.63	0.75	0.60	0.38	0.97	0.36	0.88
a96369	Pearson Correlation	0.13	0.25	0.35	0.22	0.14	0.22	0.28	−0.07	0.27	−0.05
	Sig. (2-tailed)	0.78	0.59	0.45	0.64	0.77	0.64	0.55	0.89	0.56	0.91
*t*-test	Pearson Correlation	0.24	0.08	−0.18	0.06	0.04	−0.03	0.00	−0.11	−0.03	−0.14
	Sig. (2-tailed)	0.60	0.87	0.69	0.90	0.94	0.96	10.00	0.82	0.96	0.77
CIKCAK	Pearson Correlation	0.32	0.16	−0.07	0.05	0.01	−0.03	0.19	−0.07	0.17	−0.08
	Sig. (2-tailed)	0.49	0.73	0.88	0.92	0.99	0.95	0.69	0.89	0.72	0.87
CIKCAKLop	Pearson Correlation	−0.21	−0.22	−0.52	−0.14	−0.14	−0.19	−0.53	−0.13	−0.54	−0.19
	Sig. (2-tailed)	0.66	0.63	0.24	0.77	0.77	0.68	0.22	0.77	0.21	0.68
a45	Pearson Correlation	0.25	0.17	−0.05	0.04	0.01	−0.02	0.16	−0.12	0.12	−0.15
	Sig. (2-tailed)	0.58	0.72	0.92	0.93	0.98	0.96	0.74	0.79	0.80	0.75
a45Lop	Pearson Correlation	0.18	−0.02	−0.32	0.09	0.06	0.00	−0.20	−0.11	−0.20	−0.12
	Sig. (2-tailed)	0.70	0.96	0.49	0.85	0.89	0.99	0.66	0.82	0.67	0.80
YY	Pearson Correlation	0.28	0.09	0.00	−0.12	−0.30	−0.23	0.43	0.35	0.40	0.35
	Sig. (2-tailed)	0.54	0.86	10.00	0.80	0.52	0.62	0.34	0.44	0.37	0.44

**Table 5:** Pearson’s correlation between body composition and specific parameters of motor ability in midfielders.

		BH	BW	BMI	BFkg	BF%	FMI	NFBMkg	PNTM	MMkg	PMM
CMJ	Pearson Correlation	−0.51	0.02	0.46	0.17	0.19	0.32	−0.10	−0.19	−0.08	−0.15
	Sig. (2-tailed)	0.30	0.97	0.36	0.76	0.72	0.54	0.85	0.72	0.89	0.78
CMJa	Pearson Correlation	−0.40	−0.69	−0.21	0.24	0.61	0.39	−0.95	−0.61	−0.97	−0.66
	Sig. (2-tailed)	0.44	0.13	0.69	0.65	0.20	0.44	0.00	0.20	0.00	0.15
SJ	Pearson Correlation	0.09	0.38	0.23	−0.23	−0.44	−0.25	0.60	0.44	0.63	0.49
	Sig. (2-tailed)	0.86	0.46	0.67	0.66	0.39	0.64	0.21	0.39	0.19	0.33
s20	Pearson Correlation	−0.24	−0.37	−0.10	0.20	0.39	0.24	−0.56	−0.40	−0.56	−0.41
	Sig. (2-tailed)	0.65	0.48	0.85	0.71	0.44	0.65	0.25	0.44	0.25	0.42
s505	Pearson Correlation	−0.30	−0.30	0.02	0.57	0.80	0.62	−0.77	−0.81	−0.80	−0.84
	Sig. (2-tailed)	0.56	0.56	0.97	0.24	0.06	0.19	0.07	0.05	0.06	0.04
a96369	Pearson Correlation	−0.66	−0.03	0.55	0.76	0.88	0.87	−0.61	−0.88	−0.62	−0.88
	Sig. (2-tailed)	0.15	0.95	0.26	0.08	0.02	0.02	0.20	0.02	0.19	0.02
*t*-test	Pearson Correlation	0.00	0.18	0.15	0.61	0.60	0.50	−0.26	−0.60	−0.29	−0.62
	Sig. (2-tailed)	10.00	0.73	0.79	0.20	0.20	0.31	0.62	0.21	0.58	0.19
CIKCAK	Pearson Correlation	−0.67	−0.28	0.35	0.36	0.54	0.54	−0.59	−0.54	−0.58	−0.54
	Sig. (2-tailed)	0.15	0.59	0.50	0.49	0.27	0.27	0.22	0.27	0.22	0.27
CIKCAKLop	Pearson Correlation	−0.20	−0.66	−0.36	−0.14	0.16	0.00	−0.64	−0.16	−0.65	−0.21
	Sig. (2-tailed)	0.71	0.16	0.49	0.80	0.77	0.99	0.17	0.77	0.17	0.69
a45	Pearson Correlation	−0.44	0.00	0.39	0.80	0.91	0.83	−0.60	−0.91	−0.63	−0.93
	Sig. (2-tailed)	0.38	10.00	0.45	0.06	0.01	0.04	0.21	0.01	0.18	0.01
a45Lop	Pearson Correlation	−0.12	−0.41	−0.22	0.42	0.67	0.43	−0.78	−0.68	−0.81	−0.73
	Sig. (2-tailed)	0.82	0.42	0.67	0.41	0.14	0.39	0.07	0.14	0.05	0.10
YY	Pearson Correlation	−0.52	−0.29	0.22	−0.25	−0.14	−0.01	−0.13	0.14	−0.10	0.17
	Sig. (2-tailed)	0.29	0.58	0.68	0.63	0.79	0.98	0.80	0.79	0.85	0.75

**Table 6:** Pearson’s correlation between body composition and specific parameters of motor ability in defenders.

		BH	BW	BMI	BFkg	BF%	FMI	NFBMkg	PNTM	MMkg	PMM
CMJ	Pearson Correlation	0.06	−0.47	−0.60	−0.60	−0.67	−0.65	0.09	0.85	−0.26	0.81
	Sig. (2-tailed)	0.90	0.29	0.15	0.16	0.10	0.11	0.84	0.02	0.58	0.03
CMJa	Pearson Correlation	0.80	0.62	0.44	0.43	0.30	0.26	0.80	0.38	0.79	0.03
	Sig. (2-tailed)	0.03	0.13	0.32	0.33	0.52	0.62	0.03	0.41	0.04	0.95
SJ	Pearson Correlation	0.41	−0.22	−0.46	−0.44	−0.57	−0.55	0.27	0.75	0.04	0.76
	Sig. (2-tailed)	0.36	0.63	0.30	0.32	0.19	0.20	0.55	0.05	0.93	0.05
s20	Pearson Correlation	0.38	−0.10	−0.32	−0.27	−0.33	−0.36	−0.01	0.06	0.07	0.42
	Sig. (2-tailed)	0.40	0.83	0.49	0.56	0.48	0.43	0.98	0.90	0.88	0.35
s505	Pearson Correlation	0.31	0.78	0.84	0.85	0.87	0.85	0.57	−0.18	0.65	−0.77
	Sig. (2-tailed)	0.50	0.04	0.02	0.02	0.01	0.02	0.18	0.70	0.12	0.04
a96369	Pearson Correlation	−0.24	0.38	0.59	0.57	0.65	−0.34	−0.02	−0.54	0.14	−0.77
	Sig. (2-tailed)	0.61	0.40	0.17	0.18	0.12	0.51	0.97	0.21	0.77	0.04
*t*-test	Pearson Correlation	0.50	0.46	0.34	0.39	0.35	0.93	0.54	0.20	0.50	−0.21
	Sig. (2-tailed)	0.25	0.30	0.45	0.39	0.45	0.01	0.21	0.67	0.26	0.65
CIKCAK	Pearson Correlation	0.67	0.67	0.54	0.56	0.51	0.91	0.67	0.10	0.74	−0.26
	Sig. (2-tailed)	0.10	0.10	0.21	0.19	0.25	0.01	0.10	0.84	0.06	0.57
CIKCAKLop	Pearson Correlation	0.08	0.44	0.50	0.53	0.58	0.84	0.27	−0.19	0.30	−0.61
	Sig. (2-tailed)	0.87	0.33	0.25	0.22	0.17	0.04	0.56	0.69	0.52	0.15
a45	Pearson Correlation	0.49	0.56	0.47	0.51	0.49	0.45	0.53	0.05	0.56	−0.35
	Sig. (2-tailed)	0.26	0.20	0.29	0.24	0.27	0.31	0.22	0.92	0.19	0.44
a45Lop	Pearson Correlation	0.10	0.74	0.89	0.87	0.92	0.92	0.53	−0.16	0.55	−0.85
	Sig. (2-tailed)	0.84	0.06	0.01	0.01	0.00	0.00	0.22	0.74	0.20	0.02
YY	Pearson Correlation	−0.47	−0.89	−0.90	−0.92	−0.91	−0.89	−0.68	0.16	−0.77	0.81
	Sig. (2-tailed)	0.29	0.01	0.01	0.00	0.01	0.01	0.09	0.73	0.04	0.03

## Data Availability

Not applicable.
